# Mutational Patterns Observed in SARS-CoV-2 Genomes Sampled From Successive Epochs Delimited by Major Public Health Events in Ontario, Canada: Genomic Surveillance Study

**DOI:** 10.2196/42243

**Published:** 2022-12-22

**Authors:** David Chen, Gurjit S Randhawa, Maximillian PM Soltysiak, Camila PE de Souza, Lila Kari, Shiva M Singh, Kathleen A Hill

**Affiliations:** 1 Department of Biology Western University London, ON Canada; 2 School of Mathematical and Computational Sciences University of Prince Edward Island Charlottetown, PE Canada; 3 Department of Statistical and Actuarial Sciences Western University London, ON Canada; 4 School of Computer Science University of Waterloo Waterloo, ON Canada

**Keywords:** SARS-CoV-2, COVID-19, Ontario, virus, genetics, evolution, selection, mutation, epidemiology, variant

## Abstract

**Background:**

The emergence of SARS-CoV-2 variants with mutations associated with increased transmissibility and virulence is a public health concern in Ontario, Canada. Characterizing how the mutational patterns of the SARS-CoV-2 genome have changed over time can shed light on the driving factors, including selection for increased fitness and host immune response, that may contribute to the emergence of novel variants. Moreover, the study of SARS-CoV-2 in the microcosm of Ontario, Canada can reveal how different province-specific public health policies over time may be associated with observed mutational patterns as a model system.

**Objective:**

This study aimed to perform a comprehensive analysis of single base substitution (SBS) types, counts, and genomic locations observed in SARS-CoV-2 genomic sequences sampled in Ontario, Canada. Comparisons of mutational patterns were conducted between sequences sampled during 4 different epochs delimited by major public health events to track the evolution of the SARS-CoV-2 mutational landscape over 2 years.

**Methods:**

In total, 24,244 SARS-CoV-2 genomic sequences and associated metadata sampled in Ontario, Canada from January 1, 2020, to December 31, 2021, were retrieved from the Global Initiative on Sharing All Influenza Data database. Sequences were assigned to 4 epochs delimited by major public health events based on the sampling date. SBSs from each SARS-CoV-2 sequence were identified relative to the MN996528.1 reference genome. Catalogues of SBS types and counts were generated to estimate the impact of selection in each open reading frame, and identify mutation clusters. The estimation of mutational fitness over time was performed using the Augur pipeline.

**Results:**

The biases in SBS types and proportions observed support previous reports of host antiviral defense activity involving the SARS-CoV-2 genome. There was an increase in U>C substitutions associated with adenosine deaminase acting on RNA (ADAR) activity uniquely observed during Epoch 4. The burden of novel SBSs observed in SARS-CoV-2 genomic sequences was the greatest in Epoch 2 (median 5), followed by Epoch 3 (median 4). Clusters of SBSs were observed in the spike protein open reading frame, ORF1a, and ORF3a. The high proportion of nonsynonymous SBSs and increasing dN/dS metric (ratio of nonsynonymous to synonymous mutations in a given open reading frame) to above 1 in Epoch 4 indicate positive selection of the spike protein open reading frame.

**Conclusions:**

Quantitative analysis of the mutational patterns of the SARS-CoV-2 genome in the microcosm of Ontario, Canada within early consecutive epochs of the pandemic tracked the mutational dynamics in the context of public health events that instigate significant shifts in selection and mutagenesis. Continued genomic surveillance of emergent variants will be useful for the design of public health policies in response to the evolving COVID-19 pandemic.

## Introduction

SARS-CoV-2 is responsible for the global COVID-19 pandemic, and there have been 4,109,931 total confirmed COVID-19 cases in Canada as of August 12, 2022 [1]. As the most populated province in Canada, Ontario reported 1,394,524 confirmed COVID-19 cases and 52,998 hospitalizations as of August 13, 2022 [2], among a population estimated to be 15,007,816 during the second quarter of 2022 [3]. The adaptive evolution of more transmissible and virulent COVID-19 variants associated with different acquired mutations over time may lead to increased case counts, increased mortality rates, and reduced effectiveness of general COVID-19 vaccines [4]. The emergence of novel SARS-CoV-2 variants of concern over time may in part be attributed to both the innate error rate of SARS-CoV-2 replication and the different sources of host somatic mutagenesis that cause nonrandom patterns of mutation types and counts in viral genomes. Some known mechanisms that drive SARS-CoV-2 genomic evolution are commonly associated with host antiviral defenses, including the antiviral activity of (1) apolipoprotein B mRNA editing enzyme catalytic polypeptide-like (APOBEC) family causing C>U nucleotide substitutions, (2) reactive oxygen species (ROS) causing G>U substitutions, and (3) adenosine deaminase acting on RNA (ADAR) causing A>G and U>C substitutions [5,6]. Each host-specific antiviral defense mechanism may generate a unique set of mutation types and abundances over time, known as a mutational signature, which can be used to identify which and to what extent specific mutational processes contribute to all of the mutations observed in each genome [7]. Tracking the abundance of different substitution types over time can provide insights into the contribution of each mechanism of host antiviral defense to nucleotide changes in the SARS-CoV-2 genome [8].

Acquired mutations at specific sites in open reading frames [9] or near N6-methyladenosine (m6A) methylation sites [10] of the SARS-CoV-2 genome may confer advantages in viral transmissibility, host invasion, and reproduction, and modulate the severity of the clinical symptoms of COVID-19. Previous research has identified 8 m6A methylation sites as potential sites of negative regulation of viral infection [10]. Quantifying how the landscape of SARS-CoV-2 mutations in the SARS-CoV-2 genome changes over time can reveal how viral evolution may be associated with specific patterns of mutation burden, mutation types, and genomic locations of mutations, as well as different selection pressures. Moreover, the ratio of nonsynonymous to synonymous mutations in a given open reading frame, known as the dN/dS ratio, can be used to estimate the extent of and change in positive or negative selection of protein-coding regions across the SARS-CoV-2 genome over time.

The initial spread of COVID-19 in China was limited in part due to movement restrictions set in Wuhan in January 2020, followed by the rapid implementation of nonpharmaceutical interventions, including case isolation, physical distancing, wearing face masks, and contact tracing. These interventions were effective at reducing the seropositivity rate below the threshold for an epidemic [11]. However, international travel [12] and a reduction in nonpharmaceutical interventions due to lifting of regional mandates [12,13] have been associated with the spread of novel variants and waves of infections around the world since the initial COVID-19 outbreak in Wuhan. Similarly, changes in public health policy over the course of the pandemic may influence the transmission and emergence of new SARS-CoV-2 variants in Ontario, resulting in fluctuations in the prevalence of variants and genomic diversity reflected in the landscape of novel mutations observed in each epoch.

Previous SARS-CoV-2 genomic studies have characterized the landscape of mutation types and allele frequencies around the world, including but not limited to the United States [14], Qatar [15], the United Kingdom [16], Uruguay [17], and Canada [18]. Public health policies implemented in different countries to reduce COVID-19 transmission may in part influence the emergence of novel SARS-CoV-2 genetic variants [19] and mutation rates that can lead to the evolution of resistance to vaccines [20]. For instance, genomic epidemiology of the first 2 waves of SARS-CoV-2 in Canada revealed that the number of sublineages imported to Canada reduced by over 10-fold when restrictions of foreign nationals were implemented in March 2020. Thus, public health policies and travel restrictions can affect the number of opportunities for novel SARS-CoV-2 lineages to seed new outbreaks or challenge existing lineages. The change in SARS-CoV-2 genomic diversity in the viral population over time is attributed to both the impact of different public health policies and the introduction of novel international variants.

Similar to Canada, Spain employed a multistage nonuniform lockdown, while China employed more immediate and widespread lockdown procedures to reduce COVID-19 transmission compared with Canada [21,22]. Moreover, inconsistent provincial and territorial public health responses to the COVID-19 pandemic in Canada have been reported to be less effective at reducing COVID-19 transmission and SARS-CoV-2 genetic diversity in the Canadian population [23]. Effective epidemiological surveillance requires rigorous and reliable COVID-19 testing of case counts as well as genome sequencing to track the genomic mutations that are associated with increased transmission. A database of SARS-CoV-2 genomic sequences is necessary to track the genomic evolution of SARS-CoV-2 across the world. The Global Initiative on Sharing All Influenza Data (GISAID) is a database of influenza genomic sequences, and associated clinical and epidemiological data, which has facilitated the rapid public sharing of SARS-CoV-2–related data necessary for genomic surveillance during the COVID-19 pandemic [24].

Thus, characterizing the evolution of the SARS-CoV-2 mutational landscape in the Canadian province of Ontario as a microcosm can reveal in part how Ontario-specific public health decisions and the introduction of novel variants during different time-based epochs may in part be associated with changes in the mutational landscape. This study is the first to characterize the change in the mutational landscape of 24,244 SARS-CoV-2 genomes sampled in Ontario from January 1, 2020, to December 31, 2021, at a large scale and over time, expanding on previous studies by increasing the sample size [25] and reporting the results of SARS-CoV-2 genomic surveillance over successive epochs of time [4]. In line with these studies, we also confirm previous reports of nonneutral selection in open reading frames, such as the spike and nucleocapsid open reading frames, as well as ORF3A in a larger curated data set of SARS-CoV-2 genomes sampled from the Ontario microcosm. Moreover, genomic surveillance is useful for quantifying the diversity of different variants over time as a measure of the effectiveness of public health policies to reduce transmission [26] and make rapid policy changes as well as predict conserved genomic regions undergoing negative selection as promising targets for vaccine design [27].

The surface-exposed SARS-CoV-2 spike glycoprotein, similar to other class I viral fusion proteins, such as influenza virus haemagglutinin and HIV envelope glycoprotein, regulates viral entry into host cells by changing conformation from a metastable unliganded state to a liganded stable state [28,29]. Previous studies have suggested that the spike protein mainly binds to the angiotensin II receptor to enter host cells [30]. In studies of viral challenge by SARS-CoV with similar class I viral fusion proteins as in SARS-CoV-2, polyclonal antibody responses targeting the spike protein were effective in inhibiting viral entry and decreasing viral load [31]. The design of antiviral agents that target the spike protein, a known region of positive selection that influences viral transmission, has been proposed to target the spike protein-angiotensin converting enzyme II binding interface, the auxiliary receptors involved in viral-cell fusion, or the specific epitopes of receptor binding motifs [32].

Recently, conserved pan-variant epitopes across the Alpha, Beta, Gamma, and Epsilon variant spike proteins have been successfully targeted using a neutralizing antibody fragment [33]. The changes in the patterns of mutation types, mutation genomic locations, and selection pressures of open reading frames associated with viral fitness between successive epochs suggest that the prediction of highly confident conserved epitopes and the resulting design of therapeutics with sustained efficacy may remain a significant challenge. The design of specific therapeutic approaches and vaccines for SARS-CoV-2 requires ongoing genomic surveillance to monitor their efficacy in combatting novel as well as increasingly resistant and transmissible SARS-CoV-2 variants [34]. As the COVID-19 pandemic continues, the genomic diversity of SARS-CoV-2 in Ontario may increase owing to the emergence of novel mutations acquired in part due to the host immune response and the increasing pool of infected hosts. Moreover, selection for emergent variants with mutations that confer a fitness advantage, characterized by increased immune escape and transmissibility, may further drive the genomic evolution of SARS-CoV-2 [35,36].

Here, we compared the novel changes in the proportions of single base substitution (SBS) types and the total burden of novel SBSs from SARS-CoV-2 genomes across 4 successive epochs. We tested if observed SBSs in open reading frames cluster near genes associated with increased viral fitness or known m6A sites across 4 successive epochs. Selection using the dN/dS ratios of different coding regions and the diversity of substitution types across the whole genome was compared between the 4 epochs to identify which coding regions were likely conserved. Finally, estimations of the rates of novel SBSs and the change in mutational fitness over time were used to estimate the evolution of the SARS-CoV-2 mutational landscape in Ontario.

This comprehensive study investigating the SARS-CoV-2 mutational landscape in the Ontario microcosm provides a foundation for research into simulating genomic epidemiology parameterized using both genomic and public health factors to inform public health decision-making as well as predict the activity of mutational processes and selection pressures that drive SARS-CoV-2 genomic evolution.

## Methods

### Data Collection

Data for 24,244 complete (>29,000 base pairs), high coverage (<1% N’s), and nonduplicate SARS-CoV-2 genomic sequences in FASTA format sampled from January 1, 2020, to December 31, 2021, were retrieved from the GISAID database on January 1, 2022 [20]. Metadata associated with each genomic sequence, including sampling date and GISAID clade assignment [37], were also retrieved from the GISAID database. Duplicate sequences were removed if the FASTA header and associated metadata were the same. Whole-genome alignment of SARS-CoV-2 genomic sequences to the 29,903 base-pair reference genome (GenBank accession number MN908947.3) was conducted using MAFFT (Multiple Alignment with Fast Fourier Transform) version 7 with default parameters and keeping the alignment length between the sequence and the reference genome. Custom code was used to identify SBSs observed in aligned SARS-CoV-2 genomic sequences compared to the reference genome by iteratively checking if the base type at each position of the genomic sequence and reference genome was different, and if so, updating the data table with the observed SBS position, the reference base, and the alternate base for downstream analysis of mutational patterns. Custom code used in this study was implemented using Python version 3.8.0. The final position of each observed SBS is referenced based on the reference genome position. Minor allele frequency, defined as the proportion of the SARS-CoV-2 population sampled during a given epoch with the allele, was calculated for each minority allele at each base position.

**Figure 1:**
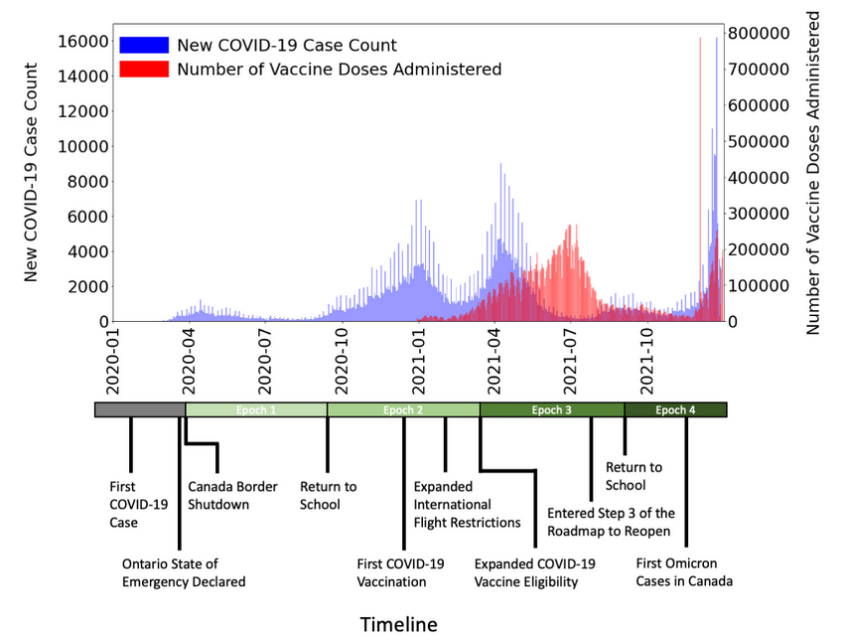
Timeline of the 4 successive epoch time periods annotated with major public health events from April 1, 2020, to December 31, 2021. The public health events are relevant in the timeline given their impact on virus spread. The number of new COVID-19 case counts (blue) and number of vaccine doses administered (red) are plotted over time. The Ontario State of Emergency Declaration (March 17, 2020) instituted a province-wide lockdown imposing severe restrictions on human behavior with the intent to greatly limit the spread of infection. Re-entry from lockdown was phased in steps through the provincial Roadmap to Reopen. Significant public health policies associated with changes in viral transmission in the timeline include the Roadmap to Reopen and return to school.

Sequences were assigned to 4 different epochs based on time as follows: Epoch 1 from April 1, 2020, to August 31, 2020 (n=2256); Epoch 2 from September 1, 2020, to February 28, 2021 (n=4443); Epoch 3 from March 1, 2021, to August 31, 2021 (n=12,864); and Epoch 4 from September 1, 2021, to December 31, 2021 (n=4102) (Figure 1). The start of each epoch closely coincided with major public health events that may be associated with reduced opportunities for viral transmission (eg, province-wide lockdowns with closure of nonessential businesses, introduction of international travel restrictions, and warmer weather) or increased viral transmission (eg, reopening of schools, lifting of international travel restrictions, and cooler weather), including the Ontario State of Emergency declaration in Epoch 1, the 2020 return to school in Epoch 2, expanded COVID-19 vaccine eligibility in Epoch 3, and the 2021 return to school in Epoch 4. Epoch-specific genomic mutations, defined as mutations in 1 epoch not observed in any previous epoch, were used for the downstream mutational pattern, clustering, selection, and diversity analyses. Mutations specific to Epoch 1 were determined by identifying mutations not previously observed in SARS-CoV-2 genomic sequences sampled from January 1, 2020, to March 31, 2020, in the collected data set (n=579).

### Ethical Considerations

We confirm that all secondary analyses of research data from the GISAID database were performed in accordance with relevant usage guidelines and regulations. The genomic data retrieved from the GISAID database were deidentified and could not be linked to patients’ identities [24].

### Generation of SBS-96 Mutational Catalogues

Each SBS for each SARS-CoV-2 genomic sequence was assigned to 1 of 96 possible classes, where each class was defined by 6 base substitutions represented by the pyrimidine of the Watson-Crick base pair (C>A, C>G, C>U, U>A, U>C, and U>G) and the flanking 5′ and 3′ bases of the SBS that forms the local trinucleotide context [7]. For example, both the C>A pyrimidine substitution and G>U purine substitution were referred to by their pyrimidine base pair, C>A. For each SARS-CoV-2 genomic sequence, a mutational catalogue of the count of each of the 96 SBSs observed was generated.

To compare the mutational catalogues between multiple sequences in each epoch, the mean proportion of each of the 96 SBS types for each epoch was generated. First, the proportion of each of the 96 SBS types was calculated by dividing the count of each of the 96 SBS types by the total count of all SBS mutations observed in each SARS-CoV-2 genomic sequence. Second, the mean proportion of each of the 96 SBS types was calculated by summing the proportion of each of the 96 SBS types across all SARS-CoV-2 genomic sequences assigned to the same epoch and dividing the sum by the number of SARS-CoV-2 genomic sequences in the epoch.

### Somatic Mutation Clustering Near Open Reading Frames and m6A Methylation Sites

The distribution of sites where novel SBSs were observed along the whole genome was analyzed to detect the presence of SBS clusters in specific open reading frames or near known m6A methylation sites [10]. Open reading frames with observed clusters of epoch-specific mutations were identified if the positions of SBSs in the open reading frame were different from a random sample of 100 positions in the open reading frame using a 2-sample Kolmogorov-Smirnov test. Eight known m6A methylation sites of the SARS-CoV-2 genome were previously identified [10]. Observed clusters of epoch-specific mutations near m6A methylation sites were identified if the positions of SBSs within a window of ±1500 base pairs of one of the m6A methylation sites were different from a random sample of 100 positions in the same window using a 2-sample Kolmogorov-Smirnov test. The ±1500 base-pair window flanking each m6A methylation site was chosen based on a previous study that first identified the m6A methylation sites and reported clustering statistics near these sites using the same ±1500 base-pair window [10].

### dN/dS Ratio of Open Reading Frames

To quantify the change in the selection pressures of each open reading frame over time, the mean dN/dS ratio of each open reading frame was compared between different epochs. First, we calculated the proportion of synonymous (pS) and nonsynonymous SBSs (pN) by dividing the count of pS and pN by the count of synonymous and nonsynonymous sites, respectively, for each open reading frame. Next, we estimated the count of synonymous (dS) and nonsynonymous (dN) substitutions per site using the Nei and Gojobori method shown in Equations 1 and 2 [38,39]. Null values of the dN/dS ratio, such as when zero nonsynonymous substitutions in an open reading frame were observed in a given SARS-CoV-2 genomic sequence, were excluded from the analyses.

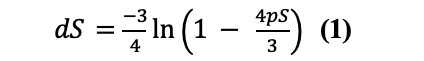

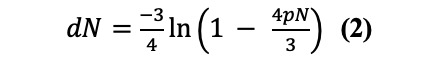





### Site-Specific Shannon Diversity Index

To compare the differences in genetic diversity at each genomic site over time, the Shannon diversity index was calculated at each genomic site for SARS-CoV-2 genomic sequences in each epoch. The mean diversity index value across the whole genome and site-specific diversity index values were compared between SARS-CoV-2 genomic sequences from different epochs [40].

### Nextstrain: Annual Rate of Novel SBSs and Estimation of Mutational Fitness

Out of 24,244 SARS-CoV-2 genomic sequences included in this study, 7398 SARS-CoV-2 genomic sequences with complete sampling dates, including day, month, and year, were used in the Nextstrain Augur 18.0.0 and Auspice 2.32.1 pipelines [41] for phylogenetic analysis and visualization, respectively [42]. The Nextstrain set of pipelines was used to estimate the rate of novel substitutions per year and the change in mutation fitness over time. Mutation fitness is defined as a metric that predicts viral reproduction and transmissibility based on the contribution of multiple somatic mutations that have been annotated to affect lineage growth, such as mutations that confer a fitness advantage by evading host immunity or decreasing generation time [43].

## Results

### Host Antiviral Defense Mechanisms are Associated With Nonrandom Biases in the Prevalence of Different SBS Types

**Figure 2:**
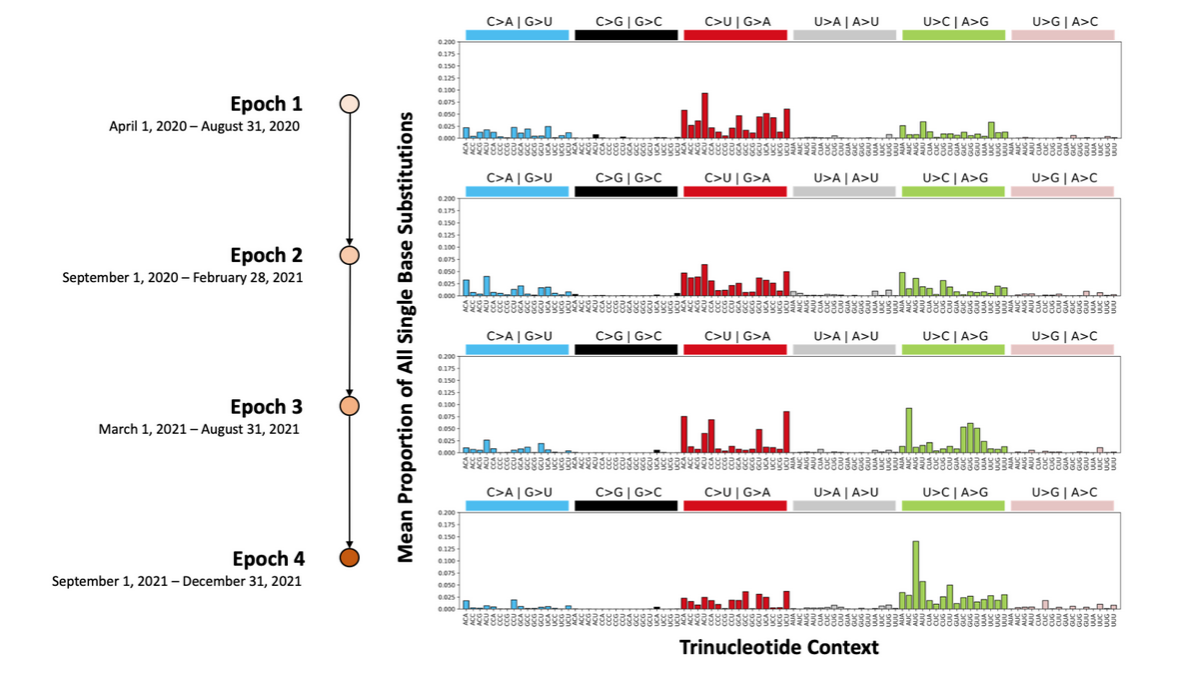
The mean proportion of single base substitution (SBS)-96 mutation types of each of the 4 different epochs. The mean proportion shows, on average across all SARS-CoV-2 genomic sequences sampled during the epoch, what proportion each SBS type makes up out of all 96 possible SBS types. At least 80% of novel SBSs in each epoch were C>U, C>A, or U>C substitutions that have previously been attributed to the activity of apolipoprotein B mRNA editing enzyme catalytic polypeptide-like, reactive oxygen species, and adenosine deaminase acting on RNA, respectively.

Patterns of mutation types and their proportions commonly associated with mutagenesis due to host antiviral defense mechanisms were quantified by classifying the novel SBSs observed in each epoch into the SBS-96 classification scheme. Next, the SBS-96 mutation types and mean proportions were compared between different epochs (Figure 2). There were 1767 unique epoch-specific SBSs in Epoch 1, while there were 3573, 4822, and 2076 such SBSs in Epochs 2, 3, and 4, respectively (Multimedia Appendix 1).

Across each of the 4 epochs, at least 80% of the novel SBSs were C>U, C>A, or U>C substitutions that have previously been attributed to the activity of APOBEC, ROS, and ADAR, respectively. Moreover, we observed that pyrimidine substitutions were more prevalent between each pair of purine and pyrimidine substitutions, constituting between 55.4% and 59.2% of all substitutions. The mean proportion of the C>A substitution type increased from 13.5% of all substitutions in Epoch 1 to 22.4% in Epoch 4. Conversely, the mean proportion of the C>T substitution type decreased from 67.9% in Epoch 1 to 48.4% in Epoch 4. The mean proportion of the T>C substitution type was relatively consistent among Epochs 1 to 3, ranging from 16.8% to 17.1% of all substitutions observed in each respective epoch. During Epoch 4, there was an increase in the mean proportion of the U>C substitution type at the AUG trinucleotide context compared to previous epochs. An increase in the mean proportion of the U>C substitution type to 19.2% of all substitutions was observed in Epoch 4 compared to Epochs 1 to 3. Visualizing each SARS-CoV-2 genomic sequence using a 3D uniform manifold approximation and projection plot of the SBS-96 mutation types and counts showed that genomic sequences sampled from the same epoch tended to uniquely cluster together (Multimedia Appendix 2).

### Average Burden of Novel SBSs Differs Between Successive Epochs

**Figure 3:**
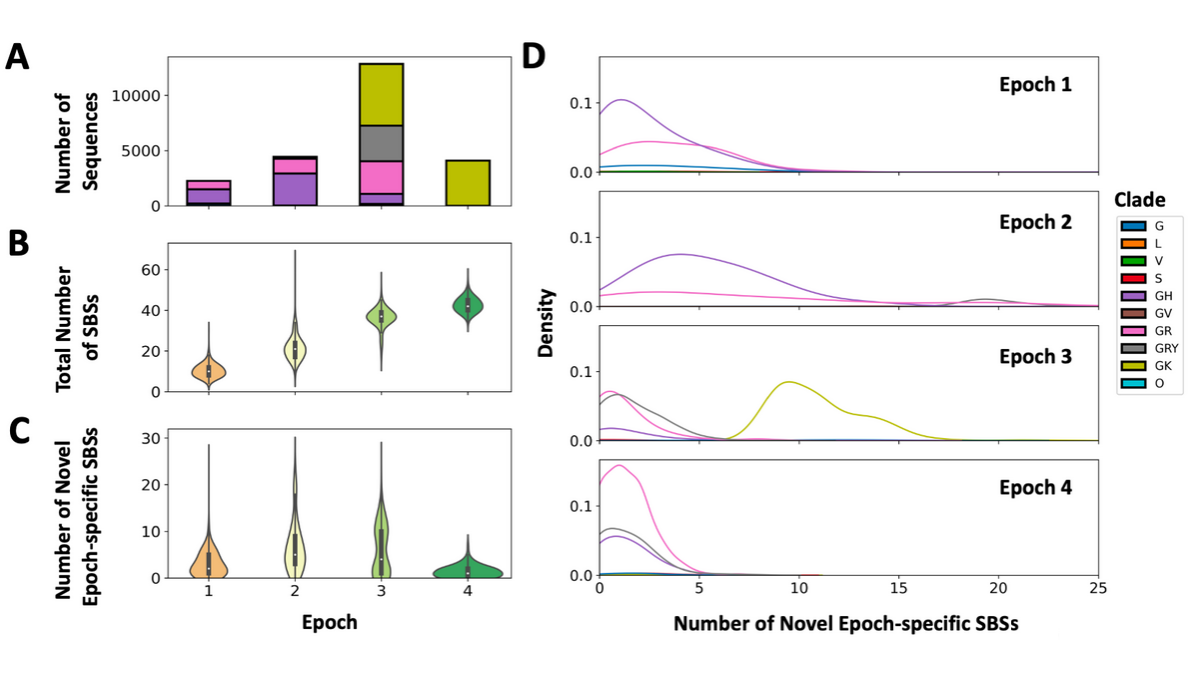
Mutational burden of single base substitutions (SBSs). Global Initiative on Sharing All Influenza Data (GISAID) clade assignments for each SARS-CoV-2 genomic sequence are based on known marker mutations associated with 8 high-level phylogenetic groupings. (A) The number of SARS-CoV-2 genomic sequences colored by GISAID clade assignment and sampled from each of the 4 epochs. (B) The distribution of the total number of SBSs observed in SARS-CoV-2 genomic sequences sampled from each of the 4 epochs. (C) The count distribution of the number of novel epoch-specific SBSs first observed in SARS-CoV-2 genomic sequences sampled from each of the 4 epochs. (D) The density distribution of the number of novel epoch-specific SBSs first observed in SARS-CoV-2 genomic sequences colored by GISAID clade assignment and sampled from each of the 4 epochs.

To analyze the variation in the number of observed mutations over time, the average cumulative SBS mutational burden and average burden of novel SBSs first observed in each epoch were compared between different epochs. Comparing the clade composition of different epochs, we observed that clade G (Delta) and GR (Gamma) sequences made up the majority of sequences sampled in Epochs 1 and 2, clade GRY (Alpha) and GK sequences made up the majority of sequences sampled in Epoch 3, and clade GK (Delta) sequences were the sole majority of sequences sampled in Epoch 4 (Figure 3A). We observed that across successive epochs, the cumulative number of observed SBSs increased as expected, with a median cumulative mutational burden of 10 SBSs in Epoch 1, 21 SBSs in Epoch 2, 37 SBSs in Epoch 3, and 41 SBSs in Epoch 4 (Figure 3B).

In addition, we observed the greatest maximum number of novel SBSs in Epoch 1 (maximum 26; median 2), followed by Epoch 2 (maximum 25; median 5), Epoch 3 (maximum 24; median 4), and Epoch 4 (maximum 8; median 1) (Figure 3C). The distribution of the mutational burden of novel SBSs in Epochs 2 and 3 was bimodal, with 2 different clade-specific populations of SARS-CoV-2 genomes with different mean counts of novel SBSs observed. In contrast, Epoch 1 and Epoch 4 showed a unimodal distribution of novel SBSs across all genomes sampled during the time period.

In Epoch 2, SARS-CoV-2 genomes from the GRY clade reported a higher burden of novel SBSs (median 19), while GH and GR clade genomes reported a lower burden of novel SBSs (GH median 5; GR median 5) (Figure 3D). In Epoch 3, SARS-CoV-2 genomes from the GR, GRY, and GH clades reported a lower burden of novel SBSs (GR median 1; GRY median 1; GH median 1), while GK clade genomes reported a higher burden of novel SBSs (median 10) (Figure 3D).

### Clusters of Novel SBSs and Selection of Open Reading Frames may be Associated With Viral Fitness

**Figure 4:**
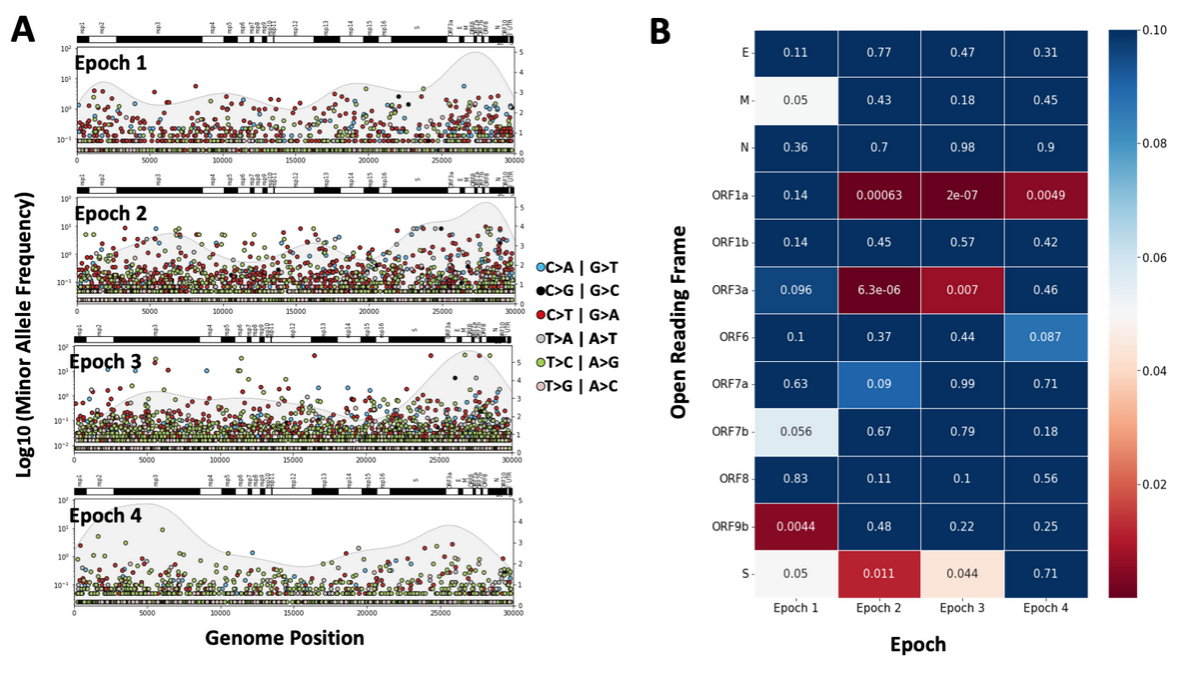
Minor allele frequency and clustering of novel epoch-specific single base substitutions (SBSs). (A) Scatterplot of the base position and the log-transformed minor allele frequency of novel epoch-specific SBSs first observed in each of the 4 epochs. The annotation of the SARS-CoV-2 genomic elements is shown above each plot. (B) Heatmap of the Kolmogorov-Smirnov test *P* value comparing the base positions of novel epoch-specific SBSs observed in each open reading frame of the SARS-CoV-2 genome and 100 randomly sampled base positions in the open reading frame, colored by *P* value (*P*<.05 is shown in red). Clusters of epoch-specific SBSs were observed in the spike protein, ORF1a, and ORF3a open reading frames during multiple epochs.

To identify open reading frames on SARS-CoV-2 genomes sampled in Ontario that may be associated with viral fitness, we observed the presence of clusters of novel SBSs in specific open reading frames compared between different epochs (Figure 4A). We consistently observed clusters of epoch-specific SBSs in the spike protein during Epochs 1, 2, and 3, but not during Epoch 4. Clusters of epoch-specific SBSs were observed in ORF1a during Epochs 2, 3, and 4. Likewise, clusters of epoch-specific SBSs were observed in ORF3a during Epochs 2 and 3, but not Epoch 4.

**Figure 5:**
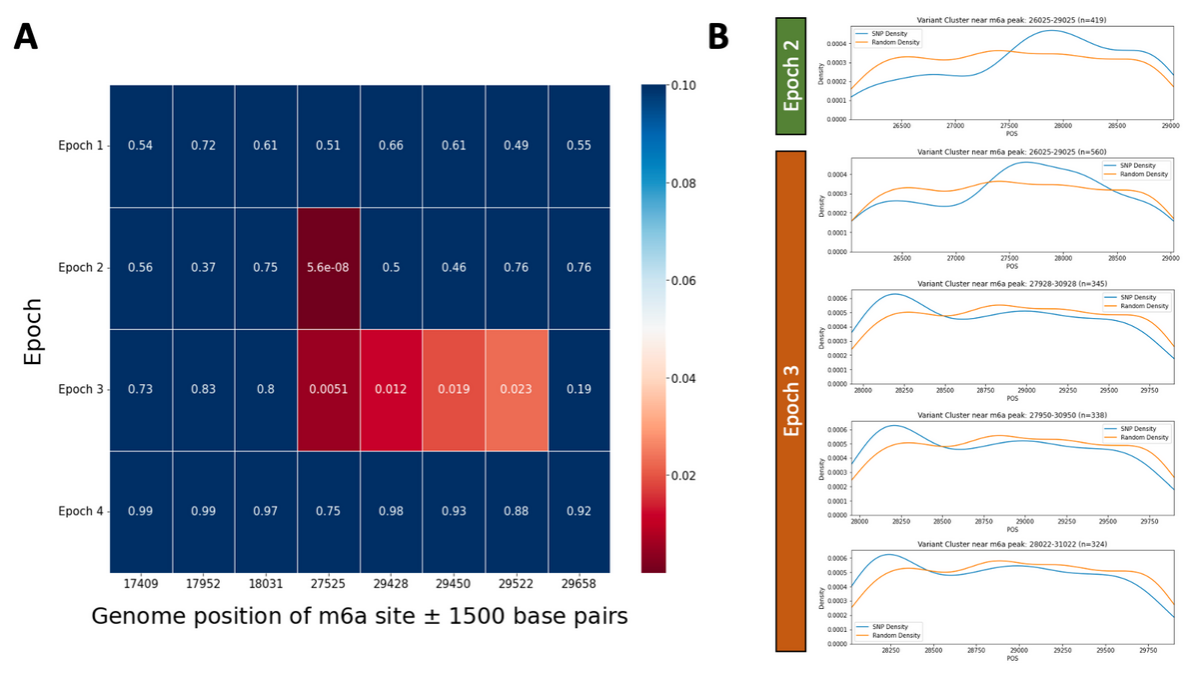
Clustering of novel epoch-specific single base substitutions (SBSs) within a ±1500 base-pair window around 8 N6-methyladenosine (m6a) methylation sites identified in the SARS-CoV-2 genome. (A) Heatmap of the Kolmogorov-Smirnov test *P* value comparing the base positions of the novel epoch-specific SBSs observed in each ±1500 base-pair window of 8 m6a methylation sites and 100 randomly sampled base positions in the same window, colored by *P* value (*P*<.05 is shown in red). (B) Density distribution of the novel epoch-specific SBSs (blue) and 100 randomly sampled base positions (orange) in the ±1500 base-pair window of 1 m6a methylation site in Epoch 2, and 4 m6a methylation sites in Epoch 3. The density distribution of SBSs is shown for 4 unique m6a methylation sites across 2 epochs since potential clusters of SBSs were detected within the ±1500 base-pair window around the m6a methylation sites.

In addition, we tested for the existence of epoch-specific clusters of novel SBSs compared to a randomly sampled distribution of 100 substitution positions within a window of ±1500 base pairs of each of the 8 known m6A methylation sites using the 2-sample Kolmogorov-Smirnov test. In Epoch 2, there was 1 m6A methylation site at position 27525 in ORF6 of the SARS-CoV-2 reference genome with a significant cluster of novel SBSs (Figure 5A). In Epoch 3, there were 4 m6A methylation sites at position 27525 in ORF6 as well as positions 29428, 29450, and 29522 in ORF10 with a cluster of novel SBSs (Figure 5A). To confirm these findings at each of the 5 m6A methylation sites with potential clusters of novel SBSs within the window, we observed that the density of the positions of mutations did show nonrandom clustering with peaks of density that differed from the randomly sampled distribution (Figure 5B).

**Figure 6:**
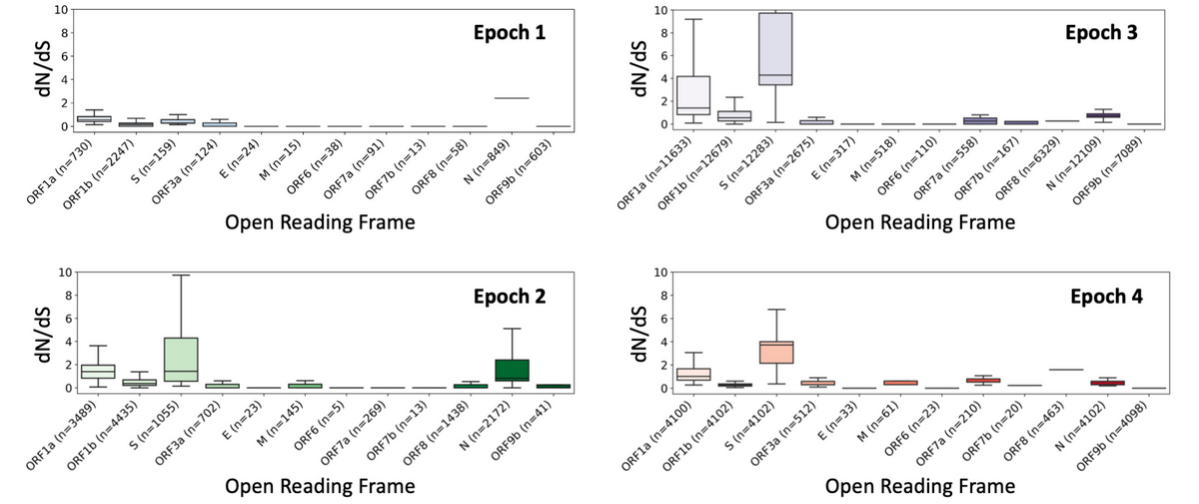
Distribution of the dN/dS metric (ratio of nonsynonymous to synonymous mutations in a given open reading frame) calculated from the novel epoch-specific single base substitutions observed in SARS-CoV-2 genomic sequences sampled during the 4 different epochs, grouped by open reading frame. The numbers of SARS-CoV-2 genomic sequences where a nonnull dN/dS metric could be calculated for the open reading frame are shown.

To quantify the selection pressures on different open reading frames of the SARS-CoV-2 genome over time, we compared the dN/dS ratio for each open reading frame between different epochs. We observed that across successive epochs, there was an increase in the median dN/dS ratio from −0.5 in Epoch 1 to 0.5 in Epoch 4 for the spike protein open reading frame (Figure 6). This suggests an increase in positive selection for nonsynonymous mutations in the spike protein. Conversely, the median dN/dS metric of ORF1b was consistently below 0 across all epochs, indicating negative selection for nonsynonymous mutations in ORF1b (Figure 6).

To characterize the diversity of different sites in the SARS-CoV-2 genome based on the different mutation types observed at the same site, we calculated the Shannon diversity index at each base position along the SARS-CoV-2 whole genome (Multimedia Appendix 3). Then, we qualitatively compared the overall profile of Shannon diversity indices between different epochs to identify if diverse mutation types tended to be observed at specific open reading frames. We found that the most recent Epoch 4 showed a relatively broad diversity across the entire genome instead of peaks of high diversity near ORF6a, ORF7a, ORF7b, ORF8, and nucleocapsid protein observed in the previous 3 epochs. Moreover, Epoch 4 most closely resembled Epoch 1 in the shared peak of site-specific diversity in nonstructural protein (NSP) 2 of ORF1a.

### Increases in SARS-CoV-2 Mutational Fitness Across Divergent Lineages Occur in Spurts

**Figure 7:**
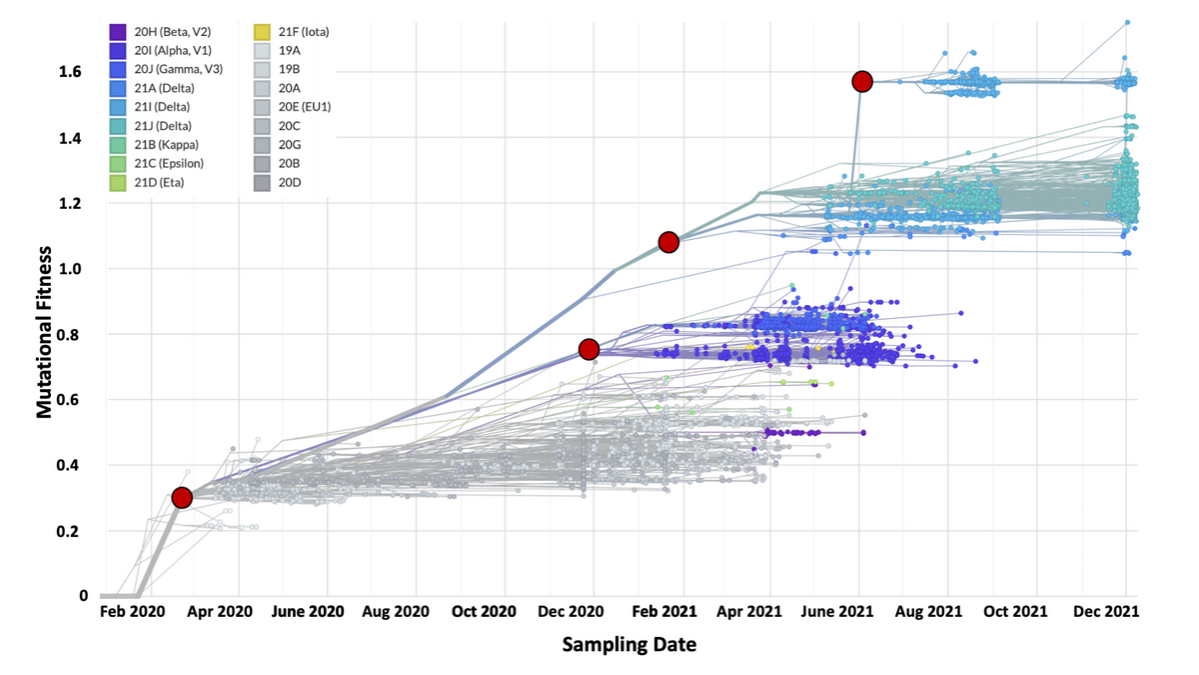
Mutational fitness of 7398 SARS-CoV-2 genomic sequences over time, colored by variant type identified using the Augur pipeline. Mutation fitness is a relative unitless metric that compares the estimated fitness of a given SARS-CoV-2 genomic sequence compared to the Wuhan reference genome, based on mutations annotated to be associated with a change in fitness. Higher mutational fitness suggests increased viral reproduction and transmissibility. Four high-level clusters of SARS-CoV-2 genomes were observed based on similarities in mutational fitness and SARS-CoV-2 variant composition. Clusters are denoted by a red marker at the earliest cluster-specific phylogenetic branch point.

To estimate the rate of novel substitutions per year and potential divergence events indicated by changes in mutational fitness, we built a rooted maximum likelihood phylogenetic tree using the Nextstrain Augur pipeline with 7398 SARS-CoV-2 genomic sequences having complete sampling dates sampled in Ontario (Figure 7). Eighteen unique clades were represented in the tree, including several variants of concern, such as Delta (21A, 21I, 21J), Gamma (20J), Alpha (20I), and Beta (20H) (Figure 6). The tree shows that the COVID-19 pandemic in Ontario is caused by multiple different lineages of SARS-CoV-2 viruses, many of which are also concurrently observed in the same epoch. The positive linear rate of acquiring novel SBSs was observed to be around 23 mutations per year.

The phylogenetic analysis revealed the timepoints of divergence of SARS-CoV-2 clades based on mutational fitness into 4 clusters. Cluster 1 was marked by an early divergence in mutational fitness in March 2020 of Epoch 1 based on branch length compared to the reference genome. Cluster 2 was predominantly composed of Alpha and Gamma variants of concern that diverged from Cluster 1 in December 2020 of Epoch 2. Following this, Cluster 3 was predominantly composed of Delta variants of concern that diverged from Cluster 2 in April 2021 of Epoch 3. Lastly, Cluster 4 was the most divergent cluster based on branch length and showed the highest mutational fitness, consisting of Delta variants of concern associated with the 20I clade observed from July 2021 in Epochs 3 and 4.

## Discussion

In this study, we quantified the changes in the landscape of SBSs observed in SARS-CoV-2 genomic sequences sampled from 4 successive epochs in Ontario from March 2020 to December 2021 during the COVID-19 pandemic. Among sequences sampled during Epoch 1, we observed positive selection and a high median dN/dS ratio above 1 in the nucleocapsid protein–coding region. Moreover, we observed clustering of novel epoch-specific SBSs, positive selection, and higher base-specific Shannon diversity in the spike protein– and nucleocapsid protein–coding regions near the 3′ end of the SARS-CoV-2 genome. During Epoch 3, we observed that there was a bimodal distribution of the number of novel epoch-specific SBSs in the GK clade and a lower number of novel epoch-specific SBSs in the GH, GV, and GRY clades. Similar to Epoch 2, there was a higher prevalence of novel SBSs in the spike protein and ORF3a-coding regions as well as positive selection of ORF1a and the spike protein–coding region. Finally, we uniquely noted that there was an increase in the mean proportion of the U>C substitution type up to 19.2% of all substitutions, especially at the AUG trinucleotide context, in Epoch 4 compared to previous epochs.

We observed that the open reading frames of SARS-CoV-2 viruses, including ORF1a and the spike protein, were increasingly impacted by positive selection over time, and this was in part consistent with mutations associated with host antiviral defenses. Future therapies can target these regions of positive selection since they affect the fitness of the SARS-CoV-2 virus [44]. Previous studies have similarly observed positive selection in the SARS-CoV-2 spike protein open reading frame [44,45] and ORF1a [46,47]. SARS-CoV-2 ORF1a and ORF1b genomic RNAs are translated and cleaved into 16 NSPs [48] associated with viral genomic replication as well as suppression of host immune response and gene expression [49]. For example, the NSP 7, 8, and 12 complex forms viral replicase machinery [50,51] and the NSP 10-16 complex is involved with capping viral mRNA transcripts and immune response evasion [52]. Due to the broad function of multiple NSPs translated from ORF1a for viral replication and the modulation of host immune response, future research to screen or design novel pharmaceutical drugs that target specific NSPs may show promise [53]. However, novel nonsynonymous mutations associated with positive selection affecting ORF1a and spike coding regions may not be conserved over time, so new iterations of pharmaceutical drugs, vaccines, or antigen tests targeting these regions may be required to maintain specificity and efficacy.

In Ontario, Epoch 1 marked the start of the pandemic from when the Canadian borders were shut down in late March 2020 to the return to school in October 2020. Similar to other regions around the world, such as the Baltimore-Washington area in the United States [54], the early measures designed to reduce local transmission of COVID-19 may have been compromised in part by the introduction of COVID-19 from national and international sources as well as the interconnectedness of the Ontario region. The high median dN/dS ratio above 1 in the nucleocapsid protein–coding region suggested that positive selection for nonsynonymous substitutions drove the early genomic evolution of the nucleocapsid protein and its function in viral genome packaging, despite being generally understood as a conserved region of the coronavirus genome [55]. Likewise, the high Shannon diversity index peaks near NSP1 and NSP2, which are involved in viral gene expression [56] and RNA binding [55], respectively, as well as the 3′ end of the SARS-CoV-2 genome, indicate a high prevalence of multiple different substitution types observed at a single genomic site. Since Epoch 1 is the first time period of the COVID-19 pandemic, the negative selection observed at all other open reading frames aside from the nucleocapsid protein–coding region may be attributed to the lag between the observation of a deleterious mutation and its subsequent selective removal from the gene pool [57]. Thus, SARS-CoV-2 genomic evolution associated with open reading frames involved in viral genome packaging and the resulting modulation of immune response were likely early events during Epoch 1 in the Ontario microcosm.

Epoch 2 was the time period from the September 2020 return to school and the introduction of the first COVID-19 vaccines in December 2020 to the expansion of COVID-19 vaccine eligibility to the general public in February 2021. Previous studies have suggested that COVID-19 vaccines likely play a role in initially reducing the genomic diversity of SARS-CoV-2 [58] and that early vaccine candidates targeting the spike protein involved in viral entry would likely be therapeutically effective against SARS-CoV-2 variants in Epoch 2 [59]. SARS-CoV-2 variants associated with clades GH and GR were the majority populations observed in both Epochs 1 and 2, but the introduction of variants associated with clade GRY (UK B.1.1.7 strain) was unique to Epoch 2 [60]. Similar to Epoch 1, clustering of novel epoch-specific SBSs and high base-specific Shannon diversity near the 3′ end of the SARS-CoV-2 genome was observed. Coupled with the positive selection observed in the spike protein– and nucleocapsid protein–coding regions near the 3′ end of the SARS-CoV-2 genome, variants observed in Epoch 2 showed increased genomic diversity in regions associated with viral attachment and entry as well as RNA genome packaging [61]. As time progressed in Epoch 2, selection pressure against SARS-CoV-2 variants with relatively decreased transmission, rate of replication, or immune defense evasion may have driven genomic evolution in favor of variants with novel mutations associated with increased fitness. Taken together, the introduction of vaccines targeting the spike protein is consistent with selection for novel mutations in the spike protein open reading frame introducing less virus susceptibility to vaccine-induced immune responses during Epoch 2 in the Ontario microcosm.

Epoch 3 spanned from March 2021 following the expansion of COVID-19 vaccine eligibility in Ontario to the September 2021 return to school. By August 5, 2021, 72% of Canadians had received 1 or more doses of a COVID-19 vaccine and 61% of Canadians were fully vaccinated with 2 doses, which were comparable to vaccination statistics observed in Ontario by this time [62]. Moreover, there was increasing demand on intensive care unit resources, and implementation of stay-at-home orders in Ontario as well as federal-mandated COVID-19 testing and 14-day quarantine of international air travelers to Canada were new measures to reduce COVID-19 case counts [62]. The emergence of GK clade SARS-CoV-2 variants with relatively high counts of novel epoch-specific SBSs and GH, GV, and GRY clade SARS-CoV-2 variants with relatively lower counts of novel SBSs comprised the bimodal distribution in the number of novel epoch-specific SBSs across all variants sampled in Epoch 3. Moreover, Epoch 3 variants were characterized by a relatively high prevalence of novel SBSs observed in the spike protein– and ORF3a-coding regions similar to Epoch 2. Likewise, both ORF1a and the spike protein–coding region showed a dN/dS ratio above 1, suggesting continued positive selection associated with nonsynonymous mutations in genomic regions involved with viral transmission and immune evasion. Moreover, the peak in the site-specific Shannon diversity index near the 3′ end of the SARS-CoV-2 genome indicates that the nonsynonymous mutations in the spike protein–coding region are diverse in the observed alternate base at each mutation site. Population mixing among vaccinated and unvaccinated populations is consistent as a contributor to the increased infection rates among vaccinated individuals than expected in a fully vaccinated population [63]. Therefore, the observed positive selection associated with novel mutations in genomic regions with impact on SARS-CoV-2 transmission and immune evasion may have been driven in part by viral evolution in the human host population with variable immune responses due to the heterogeneity of individual vaccination statuses.

Epoch 4 was from the September 2021 return to school to the end of December 2021. This epoch followed the start of Step 3 of the Roadmap to Reopen, allowing for increased numbers of people at indoor and outdoor gatherings and increased capacity at nonessential venues with the requirement of face coverings in indoor settings [64]. The first case of the emergent SARS-CoV-2 Omicron variant was identified on November 22, 2021, during Epoch 4 [65]. Interestingly, we noted that there was an increase in the proportion of U>C substitutions in SARS-CoV-2 genomes sampled from Epoch 1 to Epoch 4. Our finding is consistent with a previous report of ADAR-induced editing of A>G and complementary T>C substitutions as mutations observed more commonly in genomes sampled from late 2020 onwards [66]. Moreover, the degree of RNA deamination has been reported as a potential determinant of viral immunogenicity and infectivity in emergent minor viral populations, warranting further investigation into RNA deamination as the main driver of SARS-CoV-2 genomic evolution [66]. Compared with Epoch 3, the lower median number of novel epoch-specific SBSs observed in Epoch 4 variants and the lower dN/dS ratio of the spike protein–coding region may be due to a combination of the shorter time period, a decrease in the mutation rate, and a reduction in positive selection pressure for nonsynonymous mutations. SBSs unique to Epoch 4 were clustered in ORF1a, namely NSP2- and NSP3-coding regions associated with viral replication, and were predominantly nonsynonymous mutations as evidenced by a dN/dS ratio above 1. These findings confirm a previous report of positive selection driving the genomic evolution of NSP2 and NSP3, and the high transmissibility of COVID-19 [67]. Compared to previous epochs, the marked increase in nonsynonymous mutations and relatively higher dN/dS ratio above 2 in the NSP8 region of variants sampled in Epoch 4 may be potential mechanisms for increasing stability of the SARS-CoV-2 viral replication and transcription complex [68]. Further research is required to determine the set of genomic mutations unique to Omicron variant genomes that may provide further insights into its mechanisms of increased transmissibility, immune evasion, and decreased pathogenicity [69]. The mutation fitness of SARS-CoV-2 genomic sequences sampled in Ontario was observed to increase in spurts over short time periods, likely coinciding with the introduction of novel SARS-CoV-2 variants with acquired genomic mutations that confer a fitness advantage [70]. Thus, future research predicting the functional impact of different sets of mutations on fitness could improve the surveillance of emergent SARS-CoV-2 variants for public health and inform the design of specific antiviral therapies [71].

This study used specific dates associated with the enactment of government public health policies to examine subsequent epoch-specific mutational patterns in SARS-CoV-2 genomic sequences. The nature of this study does not permit assessment of causation between government public health policies and mutational patterns due to the potential for other contributing and potentially confounding factors, including regional weather patterns, time lag between instantiation of public health policy and practical implementation, and the development of natural and vaccine immunity in the population. Future studies may identify specific time periods when these additional factors are impactful and assess their association with mutational patterns.

As the COVID-19 pandemic continues, there is a possibility of co-infection with other respiratory pathogens [72], as well as reinfection or co-infection with multiple different variants of SARS-CoV-2 [73]. Moreover, successive selective sweeps caused in part by both mutations that confer increased fitness [74,75] and homologous recombination may give rise to novel variants [76], such as the BA.2 Omicron variant. Thus, further research into the clinical impacts of co-infection and reinfection with different SARS-CoV-2 strains may highlight the interplay between genomic variation and COVID-19 symptoms and severity.

Another consideration is the impact of COVID-19 seasonality due to regional differences in environmental factors, including temperature and humidity, that can influence viral transmission, the diversity of SARS-CoV-2 variants selected based on tolerance to different environmental conditions, and the resulting case counts [77]. Interestingly, the annual winter influenza peaks in case counts reduced during 2020 and 2021, suggesting that COVID-19–related public health measures may impact the seasonal transmission of other respiratory viruses [78]. Thus, the development of public health policies should take into account the variation in the seasonality of COVID-19 and other respiratory viruses so that health care systems are prepared for fluctuations in case counts. Further surveillance of SARS-CoV-2 genomic variation and transmission patterns across Ontario can inform effective public health decision-making and serve as a microcosm of the COVID-19 pandemic as the case count of the novel Omicron variant increases. Future design of specific antiviral therapeutics should consider ongoing genomic surveillance as a tool to identify candidate targets [79,80].

In summary, we uniquely noted a bimodal distribution in epoch-specific counts of SBSs in sequences sampled during Epoch 3, where there was a high count observed in GK clade sequences and a lower count observed in GH, GV, and GRY clade sequences. Moreover, we uniquely observed an increase in the mean proportion of the U>C substitution type up to 19.2% of all substitutions, especially at the AUG trinucleotide context, in Epoch 4 compared to previous epochs. We confirmed previous reports of positive selection and clustering of SBSs near or within the ORF1a-, nucleocapsid protein–, and spike protein–coding regions.

We characterized the mutational profile of 24,244 SARS-CoV-2 genomic sequences sampled from January 1, 2020, to December 31, 2021, in Ontario, Canada. Our findings highlight how SARS-CoV-2 genomic sequences sampled from different epochs harbor different patterns in mutational types, counts, and clusters that may be associated with differences in the transmissibility and virulence of SARS-CoV-2. Nonrandom biases in the abundance of different SBS types are consistent with the activity of host antiviral defense mechanisms and are in agreement with previous reports of the impact of host antiviral defense activity on the SARS-CoV-2 genome. Clusters of epoch-specific SBSs were observed in the spike protein, envelope protein, membrane protein, ORF3a, ORF6, and ORF7a open reading frames across all epochs, as well as near 4 unique m6A methylation sites during Epochs 2 and 3. Positive selection of the spike protein open reading frame, responsible for encoding the spike protein involved in viral entry, was observed. The estimated mutational fitness of SARS-CoV-2 genomic sequences increased in short-term spurts over time, suggesting that only a subset of somatic mutations confers a fitness advantage.

The microcosm of Ontario uniquely focuses on the evolution of the SARS-CoV-2 mutational profile associated with Ontario-specific public health events and policies. The mutational analysis of SARS-CoV-2 genomic sequences can in part reflect the impact of different public health policies during different epochs, such as the limiting of travel across Canadian borders in Epoch 1, and the impact on the genetic diversity of the SARS-CoV-2 viral population. This study of the mutational profile of SARS-CoV-2 in Ontario may serve as a model of the evolution of the SARS-CoV-2 mutational profile for comparison with other regions around the world that have implemented similar or different public health policies.

Further research of therapeutic agents designed to target conserved epitopes under negative selection, such as ORF1b, may shed light on how genomic surveillance can be a useful tool to inform the development of more effective antiviral therapies. Simulation tools used to project the evolution of SARS-CoV-2 genetic diversity due to somatic mutations or prediction models of COVID-19 waves may be parameterized using the mutational profiles and time points observed from SARS-CoV-2 sequences included in this study. To track the emergence of novel SARS-CoV-2 variants with reduced vaccine efficacy in the future, increased genomic surveillance is required, and it will inform public health policies associated with vaccine boosters as well as the implementation of nonpharmaceutical interventions such as wearing face masks and physical distancing.

## Appendix

Multimedia Appendix 1 The number of unique and shared single base substitutions observed in SARS-CoV-2 genomic sequences sampled from each of the 4 epochs.

Multimedia Appendix 2 Three-dimensional uniform manifold approximation and projection plot of the single base substitution-96 mutation types and counts observed in 24,244 SARS-CoV-2 genomic sequences sampled in Ontario, colored by the epoch when they were sampled. Each SARS-CoV-2 genomic sequence is represented as 1 circle marker. Lighter shades of each marker color indicate that the SARS-CoV-2 genomic sequence was sampled at an earlier timepoint during the same epoch compared to sequences colored in darker shades of the same color. For example, a SARS-CoV-2 genomic sequence sampled during April 2020 in Epoch 1 would be shown in lighter red, while a SARS-CoV-2 genomic sequence sampled during August 2020 in Epoch 1 would be shown in darker red.

Multimedia Appendix 3 Mean site-specific Shannon diversity index at each base position of the SARS-CoV-2 genome for SARS-CoV-2 genomic sequences sampled from each of the 4 epochs. Greater Shannon diversity index values at 1 base position suggest that the types of nucleotide bases are more diverse and evenly distributed between the different types compared to lower Shannon diversity values.

## Abbreviations

ADAR: adenosine deaminase acting on RNA
APOBEC: apolipoprotein B mRNA editing enzyme catalytic polypeptide-like
GISAID: Global Initiative on Sharing All Influenza Data
m6A: N6-methyladenosine
NSP: nonstructural protein
ROS: reactive oxygen species
SBS: single base substitution

## Data Availability

The open-source analysis code, sequence data sets, and output data sets are available at http://github.com/HillLab/SARS_CoV_2_Ontario_Genomic_Surveillance under the terms of the Creative Commons Attribution 4.0 International License.
