# Supplementary figures and images for: Mutational Patterns Observed in SARS-CoV-2 Genomes Sampled From Successive Epochs Delimited by Major Public Health Events in Ontario, Canada: Genomic Surveillance Study
Source: JMIR Bioinform Biotechnol. 2022 Dec 22;3(1):e42243. doi: 10.2196/42243 (PMC11135226; doi:10.2196/42243)

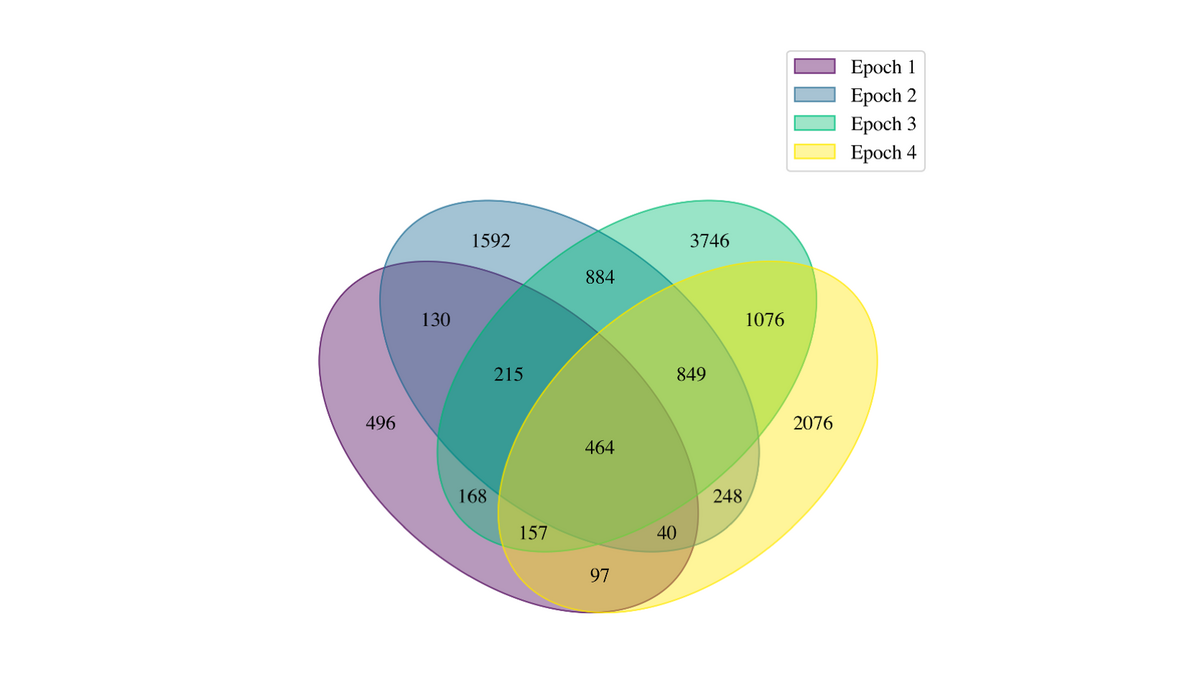

Supplement: Multimedia Appendix 1 [file bioinform_v3i1e42243_app1.png]

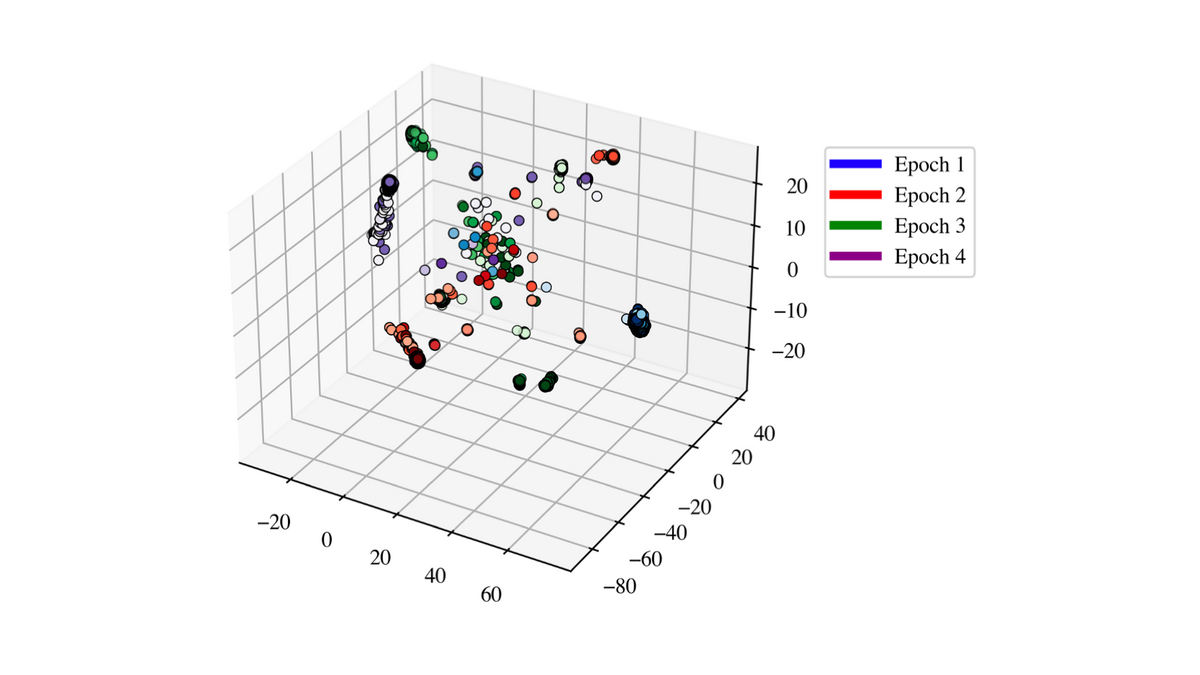

Supplement: Multimedia Appendix 2 [file bioinform_v3i1e42243_app2.png]

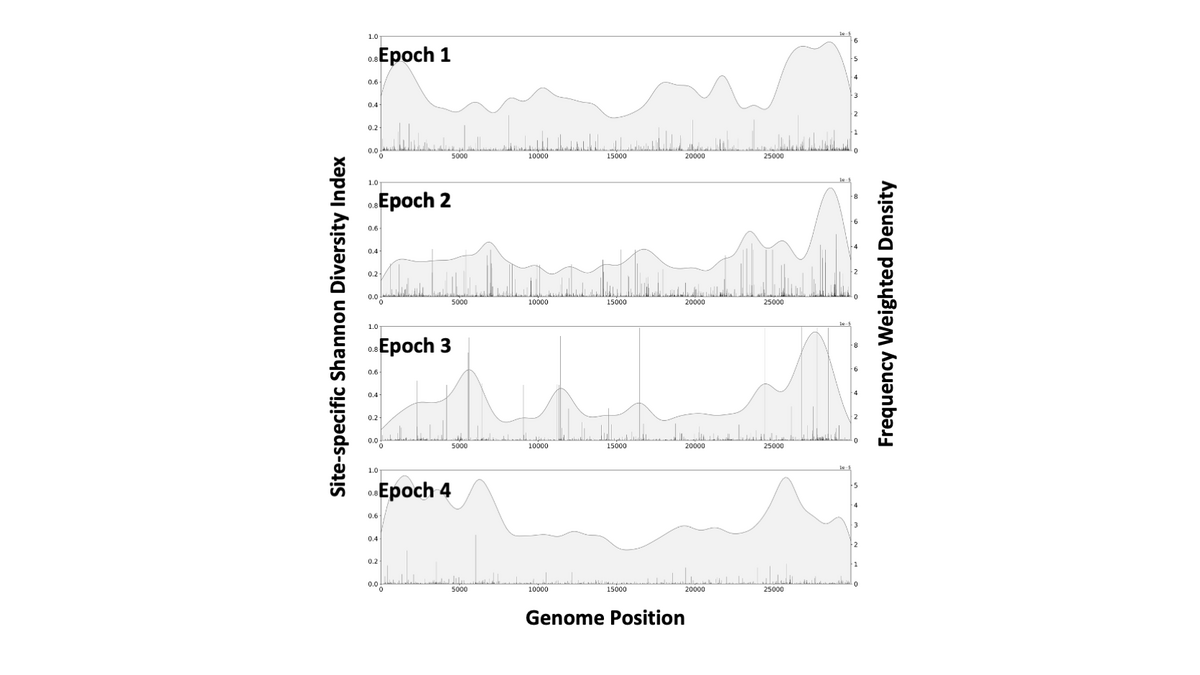

Supplement: Multimedia Appendix 3 [file bioinform_v3i1e42243_app3.png]
